# Non‐thermal technologies for broiler litter processing: Microbial safety, chemical composition, nutritional value, and fermentation parameters in vitro

**DOI:** 10.1002/vms3.1497

**Published:** 2024-07-02

**Authors:** Seyed Morteza Vaghar Seyedin, Mohsen Mojtahedi, Seyed Homayoun Farhangfar, Seyed Ehsan Ghiasi

**Affiliations:** ^1^ Department of Animal Science Faculty of Agriculture University of Birjand Birjand Iran

**Keywords:** ammonia‐nitrogen, electrocoagulation, gas production, ultrasound, ultraviolet radiation

## Abstract

**Background:**

Annually, a massive amount of broiler litter (BL) is produced in the world, which causes soil and surface water pollution due to its high nitrogen content and microbial count. While ruminants can use this non‐protein nitrogen (NPN) source for microbial protein synthesis. This issue becomes more critical when protein sources are unavailable or very expensive. One of the sources of NPN is BL which is produced at a considerable amount in the world yearly.

**Objectives:**

This aim of this research was to conduct a survey of non‐thermal technologies such as electrocoagulation (EC), ultraviolet (UV) radiation, and ultrasound (US) waves on the microbial safety and nutritional value of BL samples as a protein source in ruminant diets.

**Materials and methods:**

The methodology of this study was based on the use of an EC device with 24 V for 60 min, UV‐C light radiation (249 nm) for 1 and 10 min, and US waves with a frequency of 28 kHz for 5, 10 and 15 min to process BL samples compared with shade‐dried samples. Chemical composition and nutritional values of processed samples were determined by gas production technique and measurement of fermentation parameters in vitro.

**Results:**

Based on the results, microbial safety increased in the samples processed with the US (15 min). The EC method had the best performance in reducing the number of fungi and mould. However, none of the methods could remove total bacteria and fungi. Digestibility of BL was similar in shade‐dried, EC, and US (10 min) treatments. In general, the use of EC and US15 without having adverse effects on gas production caused a decrease in the concentration of ammonia nitrogen. In contrast, it caused a decrease in neutral detergent fibre (NDF) in the investigated substrate.

**Conclusions:**

In general, it can be concluded that the use of US5 and EC methods without having a negative effect on the parameters of gas production and fermentation in vitro, while reducing NDF, causes a significant reduction in the microbial load, pathogens, yeast, and mould. Therefore, it is suggested to use these two methods to improve feed digestibility for other protein and feed sources.

## Graphical abstract

- The 15‐min ultrasonic treatment led to a decrease in the total bacterial count and *coliforms*, as well as a decrease in the neutral detergent fibres in the broiler litter.
- The electrocoagulation and 15‐min ultrasonic treatment reduced the gas production potential and the gas production rate, respectively.
- The ammonia nitrogen and the in vitro dry matter digestibility in the broiler litter were both decreased by electrocoagulation.

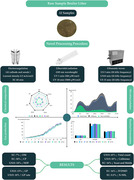

## 1 INTRODUCTION

Population growth, rising income in developing countries and increased public awareness have led to a rise in demand for food and increased consumption of animal proteins (Henchion et al., 2017). Currently, the protein requirements of livestock (ruminants and poultry) are met from two sources: human edible protein (HEP) and human inedible protein (HIP). Access to HEP due to the appropriate nutritional value of these products and the creation of food competition between humans and farm animals in the use of HEP will reduce access and price increase of these resources (Henchion et al., 2017; Te Pas et al., 2021). Increasing the efficiency of feed protein sources and converting them into animal protein (Henchion et al., 2017) and replacing HEP with the HIP (Te Pas et al., 2021) are two main strategies in managing the competition for protein sources between humans and farm animals. The rumen microbiome of ruminants gives these animals the ability to use non‐protein nitrogen (NPN) sources, after that converting them into ammonia by microbes, combined with ketoacids derived from carbohydrates and producing amino acids and in protein synthesis (Chalupa, 1978; Tadele & Amha, 2015).

So far, various HIP sources, which are all known as by‐products, such as slaughterhouse waste, feather meal, fish meal, and, broiler litter (BL) have been used as a feed in ruminants diet (Limeneh et al., 2022). The average total BL production (litter plus cake) have been reported 228.2 g dry matter (DM) material per kg live weight of broilers (g/kg) per flock (Coufal et al., 2006). BL has been replaced in ration of sheep, goat, and cattle (Daniel & Olson, 2005; Jackson et al., 2006; Obeidat et al., 2019). Considering the relatively good nutritional value of this protein source (390–430 g/kg crude protein (CP) and 181 MJ/kg energy content) (Ghaly & MacDonald, 2012), perhaps its most important advantages can be said to provide 15%–20% of ruminants needs (Van Ryssen, 2001), reduce production costs, provide minerals, and can be included in the winter and drought period (Van Ryssen, 2001). However, the use of BL in the diet of ruminants should be done with caution. As the degradable protein content in the rumen of this by‐product is high and its energy content is low, there is a possibility of large amounts of ammonia being generated in the rumen (Azizi‐Shotorkhoft et al., 2018). In such a situation, the use of high fermentable energy sources (molasses and beet pulp sugar) is recommended (Azizi‐Shotorkhoft et al., 2018). The possibility of the presence of some pathogens (Gaballah et al., 2021), toxins (mycotoxins) (Efremenko et al., 2023), heavy metals (As and Cu) (Gaballah et al., 2021), and medicine residues (antibiotics) (Efriem et al., 2023) in the BL has also been reported.

All the cases mentioned can pose a threat to humans and also endanger the health of animals. Achieving a suitable processing method to increase the digestibility of feedstuff, by‐products and agricultural residues is one of the most challenging and necessary tasks of nutritionists. All the studies conducted in BL have been using thermal processing or ensiling (Khodadadi et al., 2023). Although thermal processing reduces the microbial load, it also causes the formation of chemical toxicants in substances and the oxidation of lipids (Jadhav et al., 2021). There is another type of processing methods called non‐thermal technologies (NTTs). NTTs include electrocoagulation (EC), ultraviolet (UV) radiation and ultrasound (US) waves. NTT processing is performed at a temperature close to room temperature, and heat‐sensitive nutrients remain intact and are not harmed. NTTs have hitherto been used to process vegetables, fruit juices, meat and fish (Jadhav et al., 2021). For instance, the beneficial effects of UV radiation in the inactivating microorganisms and the reducing mycotoxin (Murata et al., 2008) and ochratoxin in feed (Ameer Sumbal et al., 2016) have been proven. Moreover, US waves have beneficial effects by creating the phenomenon of cavitation (Sunil et al., 2018). It has also been found that these waves cause changes in the protein structure (Gharibzahedi & Smith, 2020). In addition, EC has been used to remove tetracycline from livestock wastewater (Zhang et al., 2022) and the reduction lignin (Uğurlu et al., 2008; Wagle et al., 2020) and cellulose content (Jose et al., 2019).

However, studies on the application of these technologies to animal nutrition are scarce. It may be possible to modify the digestibility and nutritional value of feedstuff, particularly by‐products and agricultural residues, by employing NTTs to lower production costs and maintain quality. Therefore, the purpose of this study is to use novel processing methods such as EC, UV radiation and US waves to investigate their effects on the chemical composition, removal of pathogens and fungi, digestion and fermentation parameters in BL as an NPN source in ruminant feeding.

## 2 MATERIALS AND METHODS

### 2.1 BL preparation and processing

BL samples were obtained from an industrial chicken broiler farm in Birjand, South Khorasan province, Iran. Before applying the processing methods, the samples were dried in the shade for 72 h (control treatment or SD). Then, 10 kg of samples were ground with an industrial mill (TS‐1300) after passing through a 1‐mm sieve, and they were mixed uniformly with others. The processes applied in this study included EC, UV and US, which are discussed in detail in the following. It should be noted that all of the processing methods were performed independently in three runs with four repetitions (12 samples for each treatment).

#### 2.1.1 Electrocoagulation

**FIGURE 1:**
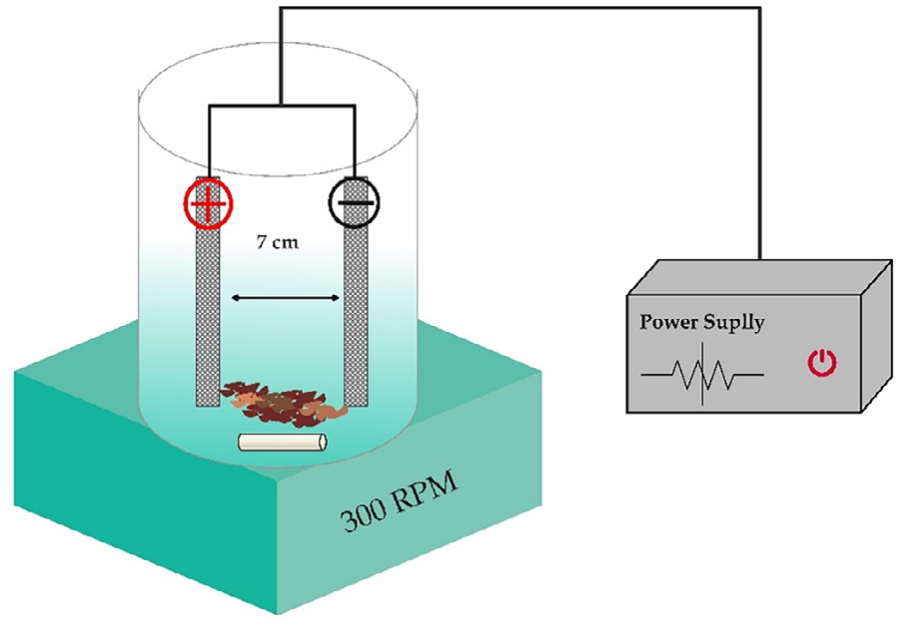
Schematic of electrocoagulation device.

An EC reactor was designed using an aluminium cathode and anode (Figure 1). Then, 25 g of the sample was added to a sterile Erlenmeyer flask containing 225 mL of sterile double distilled water (DDW), which was subjected to a voltage of 24 for 60 min. Moreover, a glass stirrer with 300 revolutions per min was used in this reactor for uniformity (Ngobeni et al., 2022). After the end of the processing time, the contents were filtered using filter paper (Whatman 42). It should be noted that the current density (*J*) in this experiment was 0.5 mA/cm^2^, which was calculated using the following equation:

$$J=I/A$$

where *I* is the current intensity (30 mA) and *A* is the surface area of the anode (60 cm^2^).

#### 2.1.2 Ultraviolet

To process with UV radiation, a UV‐C lamp (Philips, TUV PL‐S 9W/2P) with a wavelength of 249 nm was used. The samples were placed at a distance of 10 cm from the light source for 1 and 10 min. In addition, the radiation power of the used lamp was 2.3 and its radiation intensity at 1 and 10 min was 380 and 3800 μW/cm^2^, respectively (Byun et al., 2022).

#### 2.1.3 Ultrasound

Amounts of 25‐g BL were transferred into a sterile Erlenmeyer flask containing 225 mL of sterile DDW and ultrasonicated using an ultrasonic device (Parsonic 7500S, Pars Nahand) for 5, 10 and 15 min under a constant frequency of 28 kHz, and the output power was 100 W. In this section, the heat source of the device was turned off and the test was performed at room temperature. After the end of the processing time, the contents were filtered using filter paper (Shen et al., 2018).

### 2.2 Experimental treatments

#### 2.2.1 Determination of chemical composition

DM, organic matter (OM), CP, ether extract (EE) and total ash (TA) in raw and processed samples were examined using the method recommended by AOAC (2019). Moreover, contents of neutral detergent fibre (NDF), acid detergent fibre (ADF) and acid detergent lignin (ADL) were determined according to the method suggested by Jung et al. (1997) and Van Soest et al. (1991).

### 2.3 Isolation and enumeration of bacteria and fungus in BL

The method recommended by the Food Safety and Inspection Service (FSIS) was used to isolate *Salmonella* from litter samples (Gu et al., 2011; Mustafa et al., 2021). The non‐selective pre‐enrichment step was performed using a 25‐g sample and 225‐mL peptone water buffer (37°C for 24 h). Then 1 mL of the previous stage culture medium was inoculated into F broth selenite (37°C for 16 h). One loop of selenite F broth was then cultured on XLD medium and the other on McConkey agar medium (37°C for 24 h). In addition, 0.01 and 0.001 mL volumes of non‐selective pre‐enrichment medium were inoculated into McConkey agar medium for the diagnosis and isolation of *Escherichia coli* (37°C for 24 h). Possible colonies of *E. coli* with pink on McConkey agar and black colonies on XLD medium were considered possible *Salmonella* colonies. Three possible colonies from each petri dish were then used for biochemical tests, such as indole, methyl red, Voges–Proskauer and citrate (IMViC) as well as triple sugar iron agar and urease were performed to identify more accurately. Moreover, the morphologies of the colonies, such as shape, size, texture, edge, and height, colour, and turbidity formed in different media, were carefully checked and recorded.

### 2.4 Microbial count

In this study, a nutrient agar culture medium was used to count all organisms. The population of *coliforms* and fungi were counted using eosin methylene blue at 37°C for 24 h and malt extract agar (MEA) with chloramphenicol at 25°C for 3 days, respectively. All media were prepared according to the manufacturer's instructions, and 1 mL of the sample was inoculated into each culture media inside a laminar flow hood and near flame. Finally, plates containing 30–300 colonies were counted.

### 2.5 Gas production technique and ruminal fermentation and digestion parameters

The gas production technique (GPT) was performed to determine the parameters of ruminal fermentation and digestion of processed BL samples using the method proposed by Blümmel et al. (1997). For this purpose, the rumen fluid was taken from two rumen‐fistulated dry cows, which were fed according to the maintenance requirements, and placed in a flask; it was immediately transferred to the laboratory. The rumen fluid was filtered by four‐layer sterile gauze and was mixed with artificial saliva (1:2 ratio) and subjected to a continuous flow of carbon dioxide. A volume of 50‐mL mixture of rumen fluid and artificial saliva was added to 120 mL vials containing 500 mg of processed BL (with a particle size of 1 mm). Incubation was performed in a 120‐L shaker bain‐marie at a temperature of 38.6 ± 0.1°C, and the gas pressure was measured and recorded (CPG 2400 digital pressure gauge, Mensor) at times 2, 4, 6, 8, 12, 24, 36, 48, 72 and 96 h after incubation. The GPT was performed in three runs with nine repetitions at each run and three blank vials (without BL sample) to correct the produced gas. At 8 and 24 h, two vials from each treatment were randomly taken out and transferred into an ice container to stop the microbial activity. The pH of the samples was recorded by a digital pH meter (Metrohm 827 pH lab). The amount of in vitro dry matter disappearance (IVDMD) was determined by subtracting the initial amounts of DM from DM residues. Ammonia nitrogen (N‐NH_3_) concentration was evaluated using the colorimetric method (Broderick & Kang, 1980).

### 2.6 Statistical analysis

Statistical analysis of data was carried out using the PROC GLM of SAS software (version 9.4). Differences between treatments were identified using the Tukey multiple comparison test. In addition, the correlation between the variables was analysed using Pearson and Spearman correlation coefficients.

Furthermore, using the volume equation under standard pressure and temperature conditions (1 atm and 0°C), the produced gas pressure was converted into the volume of the produced gas. The gas production potential (*b*), gas production rate (*c*), and lag phase (*λ*) were calculated using the following equation (Ørskov & McDonald, 1979):

$$p\left(t\right)=b\left(1\;-\;\mathrm{ex}p^{\left[-c\left(\mathrm{time}-\lambda \right)\right]}\right)$$

## 3 RESULT

### 3.1 Chemical composition raw and processed BL

The effects of processing methods on the chemical composition of raw and processed BL samples are shown in Table 1. The processing methods caused a significant change in the amount of DM, OM, TA, CP, and NDF (*p* < 0.05). However, the content of EE, ADF and ADL was not affected by the experimental treatments. The use of EC and US methods (US5, US10 and US15) increased the moisture content in the BL samples (*p* < 0.05). In addition, EC and US methods declined OM in the samples, which was statistically significant (*p* < 0.05). BL samples processed by EC and US5, US10, as well as US15, showed a statistically significant decrease in CP compared to the UV methods and the raw sample. The amount of protein measured using the method Kjeldahl was the same in raw samples (SD) and processed with UV radiation for 1 and 10 min (UV1 and UV10) (*p* < 0.05). Moreover, the amount of NDF in BL treated with US (5, 10 and 15 min) and EC (60 min) caused a statistically significant decrease in the value of this variable (*p* < 0.05).

**TABLE 1:** Comparison of chemical composition of raw and processed broiler litter (BL) samples (*n*: 12) (mg/g).

Trait	Experimental treatments							SEM	*p*‐Value
	SD	EC	US5	US10	US15	UV1	UV10		
DM	665^a^	449^b^	478^b^	467^b^	466^b^	668^a^	666^a^	11.6147	0.0001
TA	168^b^	223^a^	211^a^	202^a^	217^a^	163^b^	164^b^	5.2746	0.0001
OM	832^a^	777^b^	789^b^	798^b^	783^b^	836^a^	835^a^	5.2746	0.0001
CP	284^a^	198^c^	218^bc^	229^b^	211^bc^	274^a^	273^a^	5.4765	0.0001
EE	14	12	13	14	13	14	13	0.4307	0.0874
NDF	348^a^	318^bcd^	302^cd^	310^d^	299^d^	337^ab^	334^abc^	5.3637	0.0009
ADF	180	173	177	181	171	176	188	5.1187	0.3193
ADL	71	70	71	70	70	71	71	0.4441	0.3694

*Note*: Means within the same row with different superscripts differ significantly (*p* < 0.05).

Abbreviations: ADF, acid detergent fiber; ADL, acid detergent lignin; CP, crude protein; DM, dry matter; EC, electrocoagulation; EE, ether extract; NDF, neutral detergent fiber; OM, organic matter; TA, total ash; US, ultrasound.

### 3.2 Microbial safety

**FIGURE 2:**
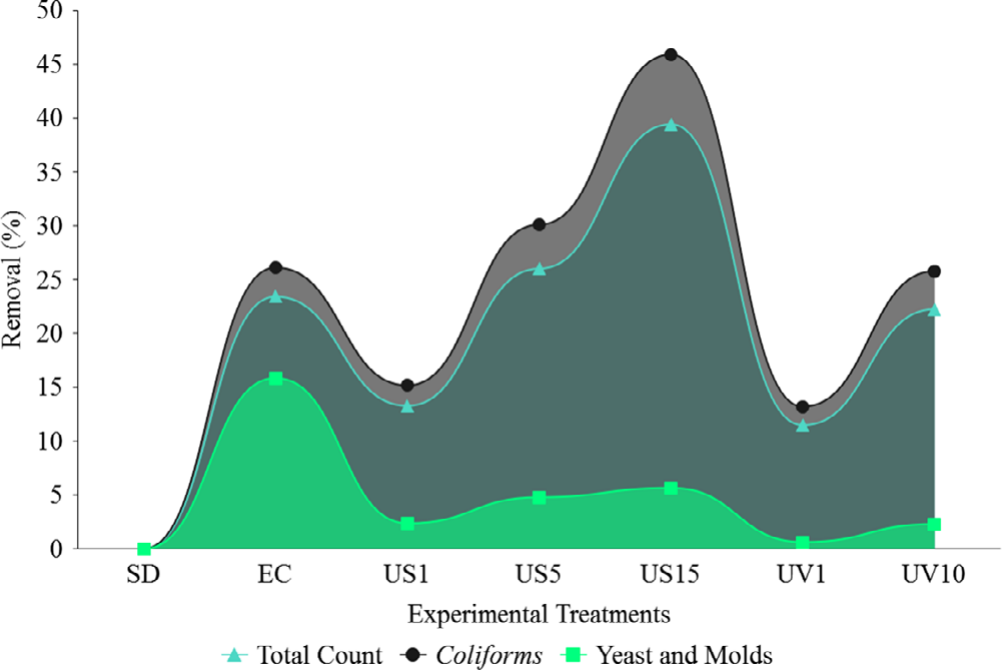
Effect of experimental treatments on the removal of the total count bacterial population, *coliforms* and yeast and molds.

The results related to the effects of processing methods on the total microbial population, *coliforms*, *E. coli*, *Salmonella* and, yeast and mould are presented in Table 2 and Figure 2. Based on the obtained information, compared to other treatments, the US15 treatment caused a significant decrease in the total bacterial, *coliforms*, and *E. coli* population (*p* < 0.05). Moreover, the use of EC, US5, US10 and US15 treatments suppressed the growth of *Salmonella* compared to other processing methods. In addition, processing with EC showed the lowest amount of yeast and mould growth, and this difference was statistically significant (*p* < 0.05). The US15 treatment resulted in the removal of 65.04% and 84.80% of the total bacterial population and *coliforms*, respectively, compared to the control treatment. Also, 13.20% and 25.78% removed *coliforms* in UV1 and UV10 treatments (respectively) compared to the control treatment (SD). In addition, the BL processed by EC caused the repression of 15.84% of yeast and moulds in BL samples. Other processing methods showed slight effectiveness (less than 6%, approximately) in removing yeast and mould.

**TABLE 2:** The effect of processing methods on the microbial and fungal population of broiler litter (BL) samples.

Trait	Experimental treatments							SEM	*p*‐Value
	SD	EC	US5	US10	US15	UV1	UV10		
Total bacteria	7.782^a^	6.306^c^	6.749^b^	5.758^e^	4.715^f^	6.889^b^	6.051^d^	0.0662	<0.0001
*Coliforms*	6.664^a^	5.284^c^	5.653^b^	4.658^e^	3.606^f^	5.784^b^	4.946^d^	0.0645	<0.0001
*Escherichia coli*	d^1^	n.d.^2^	d	n.d.	n.d.	d	d	–	–
*Salmonella*	D	n.d.	n.d.	n.d.	n.d.	d	d	–	–
Yeast and molds	4.705^a^	4.061^c^	4.594^a^	4.480^b^	4.439^b^	4.676^a^	4.597^a^	0.0367	<0.0001

*Note*: Means within the same row with different superscripts differ significantly (*p* < 0.05).

Abbreviations: d, detect; EC, electrocoagulation; n.d., non‐detect; US, ultrasound; UV, ultraviolet.

### 3.3 Gas production kinetics

**FIGURE 3:**
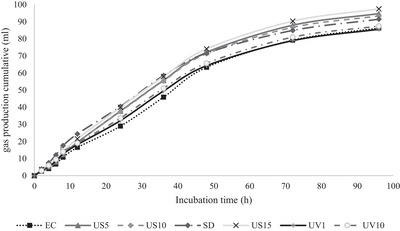
Cumulative gas production by experimental treatments.

Gas production parameters such as potential (*b*) and rate of gas production (*c*) and lag phase (*λ*) in the raw and processed BL by NTTs are shown in Table 3 and Figure 3. According to the findings, the *b* parameter in the US15 treatment decreased significantly compared to other treatments (*p* < 0.05). Also, the samples processed using US for a longer time (US15) showed a higher *c* parameter, which was statistically significant (*p* < 0.05), compared to the SD, EC and UV methods. In addition, the *λ* increased significantly the US5 treatment, but it did not differ from the EC treatment. However, the value of this variable was the same among other experimental treatments (*p* < 0.05). Also, the UV15 treatment had the lowest value *λ* among the treatments.

**TABLE 3:** Effects of different processing methods of broiler litter (BL) on gas production parameters in vitro.

Parameters	Experimental treatments							SEM	*p*‐Value
	SD	EC	US5	US10	US15	UV1	UV10		
*B*	120.200^a^	118.540^ab^	118.777^ab^	116.561^bc^	104.174^d^	114.317^c^	114.935^c^	0.8342	<0.0001
*C*	0.0197^b^	0.0151^c^	0.0192^b^	0.0192^b^	0.0232^a^	0.0181^b^	0.0183^b^	0.0004	<0.0001
*λ*	1.6008^b^	1.6940^ab^	1.9227^a^	1.6215^b^	0.6106^d^	1.4660^b^	1.0709^c^	0.0670	<0.0001

*Note*: Means within the same row with different superscripts differ significantly (*p* < 0.05). *b*, gas production potential; *c*, gas production rate; *λ*, lag phase.

Abbreviations: EC, electrocoagulation; US, ultrasound; UV, ultraviolet.

### 3.4 Ruminal fermentation and digestion parameters

The pH of the culture medium at 8 and 24 h after incubation was not affected by the processing methods. However, the application of US caused a numerical decrease in the pH of the samples after 24 h of incubation, which was not statistically significant (*p* > 0.05). In addition, the experimental treatments showed the same mean IVDMD at 8 h of incubation (*p* < 0.05). Nonetheless, the amount of IVDMD in EC treatment at 24 h was significantly lower than other experimental treatments (*p* < 0.05), but applying US and UV treatments did not change IVDMD compared to SD treatment (*p* < 0.05). However, the experimental treatments resulted in a change in the concentration of N‐NH_3_ in BL samples in vitro, and this difference was statistically significant (*p* < 0.05). The highest and lowest concentrations of N‐NH_3_ were observed in UV10 and EC treatments, respectively, but the value of this parameter was the same in SD, US5, US10, and US15 treatments, 8 h after incubation. Also, N‐NH_3_ concentration in EC treatment decreased significantly compared to other experimental treatments, after 24 h of incubation (*p* < 0.05).

### 3.5 Correlation of gas production test parameters

The results related to the correlation of gas production data, pH, IVDMD, and N‐NH_3_ concentration are shown in Table 5. Based on the analysis, there is a strong negative correlation between gas production and pH, so with increased gas production, pH decreases.

## 4 DISCUSSION

### 4.1 Chemical composition raw and processed BL

The increase in humidity in the samples processed by US and EC was due to adding of water to the samples. On the other hand, the dissolution of BL in water causes the loss of some ingredients; as a result, a decrease in the content of OM and an increase of about 6% in the content of TA in treatments processed by EC and US (US5, US10 and US15). In the present study, US methods probably caused some soluble substrates to be colloidal and removed from the BL samples after filtration, and this caused the OM content to decrease. Moreover, previous articles have proposed a chain of reactions, including hydroxylation, isomerization and demethylation to convert macromolecules into small organic compounds through free radical and non‐radical pathways by using EC as a valuable method, to remove elements, toxins and antibiotics (Zhang et al., 2022), that the particle size reduction caused more of them to leave the BL samples. As a result, the amount of minerals in the samples processed by EC treatment has decreased.

The protein content as CP was estimated in terms of the amount of total nitrogen in BL samples. Jing‐Yan et al. (2010) have reported an increase in the nitrogen content of wheat straw when exposed to UV‐B, which was not observed in the present study. According to Kumar et al. (2020), the wheat flour protein content decreased in accordance with the results of this study. As a result of radiation, nitrogen radicals are formed and released as nitrogenous gases such as ammonia (NH_3_) and nitrogen oxides. In addition, Neves‐Petersen et al. (2012) have shown that aromatic amino acids such as tryptophan, tyrosine and cysteine can cause free radical chain reactions when exposed to UV light. In addition, the release of NH_3_ due to the decomposition of proteins and amino acids under UV radiation has been reported by Kumar et al. (2020), which indicates the reduction of nitrogen content in the material. In general, the effects of UV radiation on the chemical composition of food include the reduction of vitamin content, protein breakdown, lipid oxidation and the loss of antioxidants (Csapó et al., 2019). It seems that the intensity of radiation, the type of source (UV‐ABC light), the duration of radiation exposure, the distance from the source and the nature of the product affect the CP content.

The use of EC using an aluminium electrode resulted in removing lignin, as a carbohydrate that is part of ADF (Uğurlu et al., 2008). Moreover, in another experiment, the using the EC method reduced the cellulose content of coconut fibres (Jose et al., 2019). Probably, the exposure of the fibres to the acceleration of alkali retting and its decomposition into microfibrils caused the production of fibres containing negative potential, which in reaction with sodium ions causes the formation of cellulose‐ONA and its precipitation (Jose et al., 2019). There are also reports regarding reducing various carbohydrates, such as sugar content (Chen et al., 2020) and lignin (Cogulet et al., 2016) by using UV light. However, Kumar et al. (2020) reported increased wheat flour starch by exposing it to UV light. Changes in NDF with UV1 and UV10 treatments were probably due to the free radical oxidation of hydroxide (Chen et al., 2020).

### 4.2 Microbial safety

Using EC with a current density of 0.5 mA/cm^2^ resulted in the removal of 81% of *E. coli* with an initial concentration of 10^5^ (Hashim et al., 2020b). Moreover, the results of Hashim et al. (2020a) show that the number of living *E. coli* cells decreases by 21% during 20 min of EC. In addition, Boudjema et al. (2014) have reported a 99% reduction in *coliforms* and a 100% reduction in yeast and mould by using a current density of 3 A for 60 min. There are three theories regarding the removal of microorganisms by EC. First, EC disrupts the physiological function of the cell by increasing the electrical potential difference in the cell membrane and the movement of ions (Drees et al., 2003). Second, this causes the inactivation of the living cell following the oxidation of phospholipid membrane proteins (Drees et al., 2003). Third, this brings about producing coagulant metal ions that can produce metal hydroxides (Hashim, et al., 2020a). The difference in the results is caused by the type of electrode used, the distance between the electrodes, the initial pH, the initial concentration of microorganisms and the current density.

Maryam et al. (2021) reported a 2 logarithmic reduction (LR) in mesophilic aerobic bacteria and a 17‐fold decrease in the number of total microorganisms using a US bath (frequency 40 kHz for 3 min). Also, Millan‐Sango et al. (2017) expressed 1.4 LR in the number of *Salmonella* colonies and 1.06 LR in the number of *E. coli* in alfalfa using a US probe with a frequency of 26 kHz. In another study, using a frequency of 24–26 kHz resulted in 98% inactivation of the fungus in wheat (Rudik et al., 2020). The primary mechanism of the US effect is in the inactivation of microorganisms through phenomenon of cavitation (temperature ∼5000°K and pressure >1000 atm) (Sunil et al., 2018), which forms the phenomenon of cavitation, in such a way, that it causes the formation, growth and disintegration of small bubbles in BL samples through several cycles of compression and density reduction. The disintegration of the cavitation bubble leads to releasing a significant amount of energy and induces an enormous shear force. This process leads to chemical and physical changes in bacteria and fungi structure and causes their death (Pollet & Ashokkumar, 2019; Sunil et al., 2018). In addition, it has been reported that samples with more water react more effectively to US treatment, because they cause the production of more hydroxyl radicals, hydrogen peroxide and hydrogen, which initiate the breaking of covalent bonds in molecules and reduce the number of microorganisms (Liu et al., 2019). It seems that some features of microorganisms and the characteristics of the sonication device are effective in the death of living organisms. Size, shape, cell wall, spores, growth stage and initial content of microorganisms are among the most influential factors in microorganism removal (Moosavi et al., 2021). Also, the type of sonication device (bath or probe), the device's power, intensity, current density, processing time and the properties of the foods or feeds, such as solid, liquid or semi‐solid, affect the US effects (Chemat & Khan, 2011).

The results of previous reports show 2.6 LR of *E. coli* O157:H7 by UV light for 15 min (Keklik et al., 2019). In addition, using a UVC lamp for 10 min with a dose of 774 has caused a 73% and 55% reduction in the population of *Aspergillus flavus* and *Aspergillus niger*, respectively (Begum et al., 2009). It has also been found that gram‐negative bacteria are more sensitive to UV light compared to gram‐positive bacteria (Keklik & Demirci, 2014; Keklik et al., 2019). The reason for this has been attributed to the thicker cell wall of gram‐positive bacteria, which have a more significant protective effect against UV radiation (Keklik & Demirci, 2014). The main mechanism in the inactivation of microorganisms by UV radiation is damage to deoxyribonucleic acid (DNA) and the prevention of its transcription (Brem et al., 2017). The greatest effect of UV radiation is in the wavelength of 200 to 280 nm (UV‐C) because more photons are absorbed by DNA thymine and cytosine nitrogenous bases and disrupt the transcription and replication of microorganism DNA (Brem et al., 2017; Delorme et al., 2020; Guerrero‐Beltrn & Barbosa‐C·novas, 2004). Inhibition of microorganisms is related to light intensity, distance to the source, processing time and type of fungus or bacteria (Begum et al., 2009; Delorme et al., 2020).

### 4.3 Gas production kinetics

GPT is used as an accepted method to measure the digestibility of feed ingredients in monogastric (swine and horse) (Kara, 2022) and polygastric animals (cattle, sheep and goat) (Spanghero et al., 2018). Gas production and its correction based on ammonia have been proposed as a technique to estimate the protein degradability of some feeds such as meal and forage (Raab et al., 1983). The difference in gas production in the experimental treatments indicates different digestibility in the ruminal system in vitro. Until performed this experiment, no manuscript has been published regarding the comparison among the processing methods such as EC, US and UV radiation or using any of these methods on gas production parameters. However, some reports have been made about adding BL to diets (Mirheidari et al., 2019; Sharifi & Chaji, 2019). Gas production is a quick and cheap method without toxic effects on the rumen of polygastric animals, so this section of the study was carried out with the assumption of measuring the ruminal digestibility and evaluating the processed BL on the release of ammonia. In the GPT for ruminants, about 40% of the total gas production is caused by the fermentation of the experimental feed (Menke, 1988) and the rest comes from the buffers used. For every mole of volatile fatty acid (VFA) produced, bicarbonate buffer is used and 1 mol of carbon dioxide is released (Makkar, 2005). On the other hand, a decrease in gas production per 10 g CP/kg substrate has been reported (Cone & van Gelder, 1999). The cause of this phenomenon is the reduction of gas production from buffers following the fermentation of nitrogenous compounds and the production of NH_3_ gas and the neutralization of acids (Spanghero et al., 2018). Therefore, there is a possibility that due to the increase in N‐NH_3_ production in UV1 and UV10 treatments, the *b* parameter has decreased. Also, the chemical composition of BL samples treated with different processing methods can also be a reason for the difference in degradability and then the difference in gas production (Singh et al., 2005). In addition, in this study, it was found that the samples treated with EC improved the fermentation rate and soluble substrates in the rumen fluid compared to other treatments. The use of EC processing methods may have been balanced the degradation of nitrogen and its maximum use by rumen microbes for the production of microbial protein synthesis (Azizi‐Shotorkhoft et al., 2018) and the loss of nitrogen in the form of NH_3_ is avoided (based on the results of Table 4). In the *λ*, the number of microbial cells does not increase, and in fact, it is the preparation of cells for growth and metabolic activities, and probably the increase of these parameters in EC treatment is due to the habituation of microbes to the substrate. The cause of this phenomenon is unclear, but it may be due to the chain reactions caused by the production of hydroxide and free radicals, which reasoned the increase in the *λ*. However, lower OM and NDF content (Table 1) in US15 samples can be a reason for reduced digestibility and, as a result, a decline in gas production in BL samples processed with this method.

**TABLE 4:** Effects of different processing methods of broiler litter (BL) on fermentation and ruminal digestion parameters in vitro.

Trait	Time	Experimental treatments							SEM	*p*‐Value
		SD	EC	US5	US10	US15	UV1	UV10		
pH	8	7.07	7.08	7.10	7.09	7.10	7.09	7.09	0.0089	0.2606
	24	6.58	6.52	6.48	6.46	6.45	6.53	6.54	0.0308	0.0928
IVDMD	8	27.63	26.78	27.07	27.01	27.48	27.51	27.68	0.3537	0.4528
	24	38.23^a^	32.02^b^	36.85^ab^	37.76^a^	37.11^ab^	36.73^ab^	36.48^ab^	1.0855	0.0259
N‐NH_3_	8	7.78^c^	4.73^d^	7.63^c^	7.88^c^	7.49^c^	11.85^b^	13.21^a^	0.2435	<0.0001
	24	17.33^a^	15.85^b^	17.80^a^	17.69^a^	18.36^a^	17.74^a^	17.71^a^	0.2406	<0.0001

*Note*: Means within the same row with different superscripts differ significantly (*p* < 0.05).

Abbreviations: EC, electrocoagulation; IVDMD, in vitro dry matter digestibility; N‐NH_3_, ammonia nitrogen concentration; US, ultrasound; UV, ultraviolet.

### 4.4 Ruminal fermentation and digestion parameters

After 8 h of incubation, the fermentation and production of products reaches their maximum and continue until 24 h after the start of incubation. For this purpose, times of 8 and 24 h were chosen to measure pH and IVDMD. Van Soest et al. (1991) stated that the optimal pH for microbial activity is between 6.2 and 7.2, and in the present study, the value of this parameter was between 6.45 and 7.10. The use of different carbohydrate sources and the forage to concentrate ratio have been among the most important factors influencing rumen fluid pH in previous studies (Azizi‐Shotorkhoft et al., 2018; Matra et al., 2021). Because the presence of many soluble carbohydrates brings about their fermentation by microbes and the rapid production of VFAs and lactic acid, and as a result of the accumulation of these acids, the pH decreases (Matra et al., 2021). However, the US processing method (US5, US10 and US15) numerically decreased the pH of the rumen liquid compared to other treatments, which is probably caused by the decomposition and more gas production from the substrate in these treatments (Table 3). After 8 h of incubation, IVDMD of the substrate samples was the same among the experimental treatments. After 24 h of incubation, no significant difference was observed between the experimental treatments except in the EC treatment. The reason for this reduction is not clear and it is probably due to compounds and secondary metabolites (hydroxide, chlorine, etc.) that are produced after EC process (Zhang et al., 2022). It is possible that this problem affects the microbial population and reduces digestibility. Moreover, the decrease in digestibility can be due to the decline in N content in samples treated with EC.

N‐NH_3_ concentration is an indicator of microbial protein synthesis. If the concentration of N‐NH_3_ decreases, it can be concluded that the use of N occurs in the direction of microbial protein synthesis (Chamberlain et al., 1993) and as a result, degradability increases. On the other hand, it was expected that the N‐NH_3_ concentration would be lower at 8 and 24 h after incubation, due to the lower amount of CP in EC‐treated BL samples compared SD. Moreover, UV10 treatment brought about the production of the highest amount of N‐NH_3_ in 8 h after incubation, which is probably due to the digestion of proteins and amino acids under UV radiation (Kumar et al., 2020), which may increase the access of microbes to N sources and this has resulted in an increase in the production of N‐NH_3_ in the rumen fluid in vitro.

### 4.5 Correlation of gas production test parameters

The results related to the correlation of gas production data, pH, IVDMD, and N‐NH_3_ concentration are shown in Table 5. Based on the analysis, there is a strong negative correlation between gas production and pH, so with increased gas production, pH decreases. It was also found that the increase in gas production indicates an increase in IVDMD when BL substrate is used. In addition, a significant negative correlation was observed between rumen fluid pH and IVDMD, which is caused by the decrease in degradability with pH drop in vitro. A positive correlation has been reported between gas production and digestibility with increasing rumen liquid N‐NH_3_ concentration (Khattab et al., 2013), which are in‐line with the findings of the present study.

**TABLE 5:** Pearson and Spearman correlation multiplications between gas production data and fermentation and ruminal digestion.

	Variables			
Variables	Gas production	pH	IVDMD	N‐NH_3_
Gas production	^*^	−0.9636	0.9874	0.9066
pH	−0.7788	^*^	−0.9480	−0.9073
IVDMD	0.9472	−0.7436	^*^	0.9038
N‐NH_3_	0.8285	0.7810	0.8417	^*^

* Means within same row with different superscripts differ significantly (*p* < 0.05). The right side of the asterisk is the Pearson coefficient and the left side is the Spearman coefficient.

Abbreviations: IVDMD, in vitro dry matter digestibility; N‐NH3, ammonia nitrogen concentration.

An increase in IVDMD by raising the N‐NH_3_ concentration is anticipated after nitrogen source degradation, as earlier studies have shown. Khattab et al. (2013) discovered a linear increase in DM degradability with increasing amounts of ration urea (levels of 0, 10 and 15 g/kg), which is consistent with the findings of the current study. This finding implies a positive correlation between these two variables. However, more nitrogen is present in the form of ammonia, which promotes the proliferation of rumen microbes (Boucher et al., 2007). Although Ørskov et al. (1972) observed that urea‐supplemented diets did not affect microbial protein synthesis, whereas they increased rumen fermentation and degradability. In addition, a study found that pH in diets, including date by‐product (replacement for barley grain), was negatively correlated with the concentration of N‐NH_3_ and DM degradability (Khattab et al., 2013), which is consistent with the results of this research. It has been noted that at pH values below 6.0, cellulolytic (Takizawa et al., 2020) and fibrolytic activities decline (Chiba, 2014). Additionally, the findings of Khattab and Anele (2022), which the results of this experiment support, indicated that raising the pH of rumen fluid from 5.94 to 6.31 increased the nutrients digestibility. In general, the presence of NPN sources enhances microbial protein synthesis, increasing degradability.

## 5 CONCLUSION

Using US methods (US5, US10 and US15) and EC caused the reduction of NDF in BL samples. Moreover, all the methods used reduced the total microbial load and *coliforms*. In addition, the maximum removal of pathogens (*Salmonella* and *E. coli*), yeast and mould were in the substrate treated with US for 15 min and EC, respectively. On the other hand, gas production potential was similar in US5 and EC treatments, and no change in IVDMD was observed between these two treatments. In the samples processed with EC, the reduction of N‐NH_3_ concentration was evident. In general, it can be concluded that the use of US15 and EC methods without having a negative effect on the parameters of gas production and fermentation in vitro, while reducing NDF, causes a significant reduction in the microbial count, *coliforms*, yeast and mould. Although the use of non‐thermal techniques had a positive effect on microbial safety, it did not have the same effect on digestibility as compared to the control. Therefore, it is suggested to conduct more studies to investigate these methods in other feedstuffs and by‐products.

## AUTHOR CONTRIBUTIONS

*Conceptualization; project administration; data curation; formal analysis; investigation; funding acquisition; methodology; implementation sampling; software; visualization; writing – original draft; writing – review and editing*: Seyed Morteza Vaghar Seyedin. *Methodology; supervision; funding acquisition*: Mohsen Mojtahedi. *Data curation; formal analysis; software; supervision*: Seyed Homayoun Farhangfar. *Conceptualization; investigation; methodology; writing – review and editing*: Seyed Ehsan Ghiasi.

## CONFLICT OF INTEREST STATEMENT

The authors declare no conflicts of interest.

## FUNDING INFORMATION

No grant was received for this study.

### ETHICS STATEMENT

The authors confirm that the ethical policies of the journal, as noted on the journal's author guidelines page, have been adhered to.

### PEER REVIEW

The peer review history for this article is available at https://publons.com/publon/10.1002/vms3.1497

## Data Availability

The data that support the findings of this study are available from the corresponding authors, (S.M. Vaghar Seyedin and Mohsen Mojtahedi) upon reasonable request.
